# Note from the editors: novel coronavirus (2019-nCoV)

**DOI:** 10.2807/1560-7917.ES.2020.25.3.2001231

**Published:** 2020-01-23

**Authors:** Eurosurveillance editorial team

**Affiliations:** 1 European Centre for Disease Prevention and Control (ECDC), Stockholm, Sweden

**Keywords:** public health event, coronavirus

At the end of 2019, on 31 December, the World Health Organization (WHO) Country Office in China was informed of cases of pneumonia of unknown aetiology that had been detected in Wuhan, a city in the province of Hubei. Just a day earlier, on 30 December, ProMED-mail published a post calling for more information about ‘A cluster of undiagnosed pneumonia - China (Hubei)’. The post indicated that there had been some 20 patients diagnosed with ‘atypical pneumonia’ with seven of them being severely ill [1]. In their accompanying note, the post’s moderator stated ‘*the type of social media activity that is now surrounding this event,* *is very reminiscent of the original “rumors" that accompanied the SARS-CoV outbreak*.’ [Ed*.* severe acute respiratory syndrome (SARS) coronavirus outbreak in 2003].

The situation has continued to evolve rapidly since then and just a few weeks later, as at 23 January, 614 laboratory-confirmed cases and 17 deaths have been reported [2] including some cases detected outside mainland China [3]. Meanwhile, on 7 January 2020, the novel coronavirus, currently named 2019-nCoV, was officially announced as the causative agent by Chinese authorities [3]. In order to support public health action, viral genome sequences were released by Chinese researchers on 10 January [4] and 2 days later, four further sequences were also made available on the Global Initiative on Sharing All Influenza Data (GISAID) (https://www.gisaid.org/).

While more cases are being reported on a daily basis and there is evidence for some human-to-human transmission in China, a number of important questions remain unanswered. For example, there is no certainty about the source of the outbreak, the transmissibility of the virus as well as the clinical picture and severity of the disease.

In this issue of *Eurosurveillance*, we are publishing two articles on different aspects of the newly emerged 2019-nCoV. One is a research article by Corman et al. on the development of a diagnostic methodology based on RT-PCR of the E and RdRp genes, without the need for virus material; the assays were validated in five international laboratories [5]. Before this publication, a description of the assays had already been made publically available on a dedicated WHO webpage [6] to support rapid development of laboratory testing capacities. The other is a rapid communication where researchers based in Hong Kong report on their attempt to estimate the severity among hospitalised cases of 2019-nCoV infection through modelling based on publically available information, mainly from Wuhan health authorities [7]. It also points out the need for more detailed information to make an informed evaluation of the situation as basis for public health decision-making.

Today, the WHO Director-General Tedros Adhanom Ghebreyesus, taking into consideration the deliberations of the members of the International Health Regulations (IHR) Emergency Committee on 2019-nCoV in their second meeting, decided not to declare a public health emergency of international concern.

International health organisations such as the European Centre for Disease Prevention and Control (ECDC) and the WHO are monitoring the situation and provide regular updates. ECDC has set up a dedicated webpage on which updates and risk assessments with focus on Europe are available: https://www.ecdc.europa.eu/en/novel-coronavirus-china.
